# A Systematic Review of Programs to Promote Aspects of Teen Parents’ Self-sufficiency: Supporting Educational Outcomes and Healthy Birth Spacing

**DOI:** 10.1007/s10995-019-02854-w

**Published:** 2020-01-21

**Authors:** Jessica F. Harding, Jean Knab, Susan Zief, Kevin Kelly, Diana McCallum

**Affiliations:** Mathematica, P.O. Box 2393, Princeton, NJ 08543-2393 USA

**Keywords:** Teen parents, Pregnant teens, Repeat pregnancy, Educational, Systematic review

## Abstract

**Introduction:**

Expectant and parenting teens experience many challenges to achieving self-sufficiency and promoting their children’s healthy development. Teen parents need support to help them address these challenges, and many different types of programs aim to support them. In this systematic review, we examine the research about programs that aim to support aspects of teen parents’ self-sufficiency by promoting their educational outcomes and healthy birth spacing.

**Methods:**

We conducted a comprehensive literature search of published and unpublished literature to identify studies of programs to support teen parents that met this review’s eligibility criteria. The quality and execution of the eligible study research designs were assessed to determine whether studies’ findings were at risk of bias. We then extracted information about study characteristics, outcomes, and program characteristics for studies considered to provide rigorous evidence.

**Results:**

We identified 58 eligible studies. Twenty-three studies were considered to provide rigorous evidence about either education, contraceptive use, or repeat pregnancy or birth. Seventeen of these studies showed at least one favorable effect on an outcome in one of these domains, whereas the other six did not show any significant or substantial effects in these domains. These 17 studies represent 14 effective programs.

**Discussion:**

Effective programs to support expectant and parenting teens have diverse characteristics, indicating there is no single approach for promoting teens’ education and healthy birth spacing. More rigorous studies of programs to support teen parents are needed to understand more about how to support teen fathers and the program characteristics associated with effectiveness.

**Electronic supplementary material:**

The online version of this article (10.1007/s10995-019-02854-w) contains supplementary material, which is available to authorized users.

## Significance

Teen parents face many challenges, and programs are needed to support them in achieving self-sufficiency. This systematic review illustrates that there is rigorous evidence that programs can improve teen parents’ education, increase their contraceptive use, and decrease repeat pregnancies or births. These programs have diverse characteristics, but most provide intensive, one-on-one support to teen parents. By examining a unique set of outcomes across a variety of program types, this systematic review provides useful information about the current state of the evidence regarding how to support aspects of teen parents’ self-sufficiency.

## Introduction

Becoming a teen parent can disrupt traditional life course development. Low socio-economic status and educational challenges are both predictors and consequences of giving birth as a teenager (Fergusson et al. 2007; Hodgkinson et al. 2014). Teen parents are less likely to be employed, more likely to have lower incomes, and more likely to rely on public assistance than their peers (Assini-Meytin and Green 2015; Diaz and Field 2016; Lee 2010). Nearly one in every five births to teens is a repeat birth, further heightening family disadvantage (Centers for Disease Control and Prevention 2013). These challenges are not insurmountable, but teen parents need support to address challenges, and many different types of programs aim to support them—including programs that use home visiting, group meetings of teen parents, and case management. In this systematic review, we examine the research about programs that aim to support aspects of teen parents’ self-sufficiency by promoting their educational outcomes and healthy birth spacing. This can provide useful information to the field about evidence-based models to support vulnerable teen parents.

### Educational Outcomes

Teen parents experience worse educational outcomes than teens who do not give birth during adolescence. Only half of teen mothers receive a high school diploma by age 22, compared with nearly 90% of women who have not given birth during adolescence (Perper et al. 2010). However, teen parents differ from adolescents who do not give birth during adolescence even before becoming parents—they are often more socioeconomically disadvantaged and may already be experiencing challenges in school (Coyne and D’Onofrio 2012; Xie et al. 2001). Nonetheless, rigorous studies do find negative effects of teen parenthood on education (Lee 2010). One study compared the educational attainment of sisters who gave birth at different times to determine the effect of a teen birth by using fixed effects to control for shared environmental influences (Hofferth et al. 2001). This study found that women who gave birth as teens were less likely to complete high school and some college than their sisters were. Natural experiments, which compare similar teens who get pregnant and either become teen parents or do not (based on miscarriages), also show that both teen mothers and fathers are less likely to obtain a high school diploma than are similar teens (Fletcher and Wolfe 2009, 2012).

Poor educational achievement may have subsequent effects on teen parents and their children. For example, failing to complete high school or college reduces employment opportunities and income (Bjerk 2012; Campbell 2015; Oreopolous 2008)—outcomes on which teen parents perform worse in comparison to their peers (Assini-Meytin and Green 2015; Diaz and Field 2016; Lee 2010). Lower educational attainment may also negatively affect teen parents’ children because higher parental education supports children’s academic development (Dubow et al. 2009; Harding 2015; Magnuson 2007). Indeed, some research suggests teen parenthood has negative effects on children’s development that last into their teenage years (Jutte et al. 2010; Lipman et al. 2011; Pogarsky et al. 2006; Shaw et al. 2006), although other research does not support this (Levine et al. 2007). Despite potential challenges to completing education, qualitative evidence suggests that having children may motivate teen parents to complete their education and “find a better life” (Harden et al. 2006; Herrman 2006), indicating a need to understand how programs can support teens in making educational progress.

### Healthy Birth Spacing

Having more than one child as a teen or experiencing a rapid repeat pregnancy (defined as occurring within 24 months of the prior birth) can heighten the challenges that teen parents experience as they attempt to reach their educational, career, and financial goals, and may also compound the negative effects of teen parenthood on children. Teen mothers who experience a repeat birth are even less likely to return to school, complete their high school education, or maintain economic self-sufficiency (Jones and Mondy 1994; Klerman 2004). Teen parents’ children can also experience early health difficulties from closely spaced pregnancies (Conde-Agudelo et al. 2006; Nerlander et al. 2015). For example, children who are born to teens who are already parents are at greater risk of being born early or of low birth weight (Akinbami et al. 2000), which can contribute to developmental challenges later in life (de Kieviet et al. 2009). Some research also suggests that the children of teens who are already parents are likely to experience reduced educational achievement and increased behavior problems (Klerman 2004). Although most of the research examining the effects of rapid repeat pregnancy or repeat teen births does not use methods that can demonstrate birth spacing causes negative parent and child outcomes, this research suggests that identifying evidence-based programs to promote healthy birth spacing may be important to improve outcomes for teen parents and their children (Office of Disease Prevention and Health Promotion 2014).

Teens often report their subsequent pregnancies are unintended (Herrman 2007), but contraceptive use among teen parents can be inconsistent (Boardman et al. 2006). For example, only one in five sexually active teen mothers reported using the most effective types of birth control (Centers for Disease Control and Prevention 2013). Failure to use a highly effective method of birth control is a strong predictor of a rapid repeat pregnancy (Bennett et al. 2006; Coard et al. 2000; Raneri and Wiemann 2007; Stevens-Simon et al. 2001). For example, a meta-analysis found that use of contraception, particularly long acting reversible contraceptives, reduced the risk of teens experiencing a repeat pregnancy (Maravilla et al. 2017). Therefore, identifying programs that promote contraceptive use may be a promising strategy to support teen parents’ healthy birth spacing.

### The Current Study: Identifying Evidence-Based Programs for Expectant and Parenting Teens

Given the challenges that teen parents and their children face, programs to support teen parents’ self-sufficiency are crucial (Ruedinger and Cox 2012). Teen parents are motivated to address their challenges and provide for their children (Harden et al. 2006). To help teen parents address their many interrelated challenges, the U.S. Department of Health and Human Services, Office of Population Affairs’ Pregnancy Assistance Fund (PAF) provides funds to states and tribes to develop and implement programs to improve outcomes for expectant and parenting teens. To provide information to PAF grantees as well as practitioners, researchers, and policymakers who are trying to support expectant and parenting teens, we systematically review the evidence about programs that support teen parents’ educational progression and healthy birth spacing. Increasing education and contraceptive use and reducing repeat pregnancies or births are central to the goals of the PAF program and are important aspects of promoting teen parents’ self-sufficiency (Assini-Meytin and Green 2015; Bjerk 2012; Campbell 2015; Diaz and Field 2016; Jones and Mondy 1994; Klerman 2004; Lee 2010; Oreopolous 2008). Therefore, this review provides useful information for practitioners about evidence-based programs they can use to target key self-sufficiency outcomes for teen parents. In addition, by systematically searching, rating, and then summarizing evidence across studies, this review can help stakeholders understand more about the state of the field, whether programs impact target outcomes, and the characteristics of effective programs (Paulsell et al. 2017). In this systematic review, we examine the following research questions:

1. What are the characteristics of the studies of programs to support aspects of teen parents’ self-sufficiency (education, contraceptive use, and repeat pregnancies or births)?
2. How do programs affect teen parents’ education, contraceptive use, and repeat pregnancies or births?
3. What are the characteristics of effective programs?

Past meta-analyses suggest that programs can promote positive outcomes among expectant and parenting teens, including improving teen mothers’ education (Baytop 2006; Steinka-Fry et al. 2013) and preventing subsequent births (Corcoran and Pillai 2007), although an umbrella review of these prior meta-analyses illustrated that effects were small (SmithBattle et al. 2017). In this review, we extend these past reviews by examining recent evidence about how a broad range of programs to support teen mothers and fathers affect a set of outcomes related to self-sufficiency, including education, contraceptive use, and repeat pregnancies or births. In particular, as far as we know, this is the first systematic review of programs that aim to increase teen parents’ contraceptive use. Because programs that aim to support teens may use many different approaches, such as home visiting, group-based meetings of teen parents, school-based programs, and case management, we include studies of programs that use a variety of approaches. We then look across programmatic approaches to examine the characteristics of effective programs; doing so can provide important insight for policymakers and practitioners about how to design effective programming. We also systematically assess and describe the characteristics of the existing evidence to point to next steps for future research. Overall, by examining a unique set of outcomes that support aspects of teen parents’ self-sufficiency across a variety of types of programs, this systematic review provides current evidence about how to support teen parents to promote their children’s healthy development.

## Methods

### Search Strategy

We conducted a comprehensive literature search of published and unpublished (gray) literature to identify relevant studies of programs to support expectant and parenting teen mothers and fathers. We searched in the MEDLINE, Scopus, CINAHL, PsycINFO, and ERIC databases (see Online Appendix Tables A.1 and A.2 for the full search strategy). We also searched the gray literature using Google Custom Search to create search queries to examine the entire content of selected relevant websites. Finally, to ensure a thorough literature search, we examined the reference lists of relevant review articles.

### Study Screening

A team of trained research assistants screened the titles and abstracts of all citations to identify eligible studies. Screeners were trained by the first author and screened the same set of initial studies to ensure they were applying the eligibility criteria as intended. The first author screened any studies about which the screeners raised questions. To be eligible for the review, a study had to meet the following eligibility criteria. First, it had to be a U.S. study released between 1997 and March 2017. Second, it had to evaluate a program that supports expectant and parenting teens. To allow for a broad range of programs, the review included studies of programs delivered to expectant and parenting teens using any format, including individual or group sessions and programs taking place in any type of public, private, or institutional setting. Third, a study had to use a quasi-experimental design (QED) or a randomized controlled trial (RCT) impact design to allow conclusions about the causal effectiveness of the program in relation to a comparison condition. Studies could include clusters (such as schools) or individuals. Studies without a comparison group, such as descriptive analyses or pre-post designs, were not considered. Fourth, most of the mothers and fathers in the study sample had to be younger than 21 at intake. Specifically, the review included studies in which either (1) the sample at intake was, on average, younger than 21 or (2) at least half the sample at intake was younger than 21. Fifth, the study had to examine at least one measure from three self-sufficiency-related domains: teen parents’ educational outcomes (for example, earned a high school diploma or high school grade point average); contraceptive use (for example, use of long acting reversible contraceptives or number of unprotected sex occasions); or repeat pregnancy or birth (for example, incidence of repeat pregnancy or birth). Both self-reported measures and administrative data (for example, school or birth records) were eligible for this review. Finally, studies had to include an analytic sample larger than 30 participants across the treatment and comparison groups.

### Assessing the Risk of Bias

Findings from a single study presented in multiple reports or journal articles were linked and assessed together (What Works Clearinghouse 2017). All studies that met the review eligibility criteria were assessed by two reviewers from a team of reviewers who were trained by the first author. Reviewers assessed the quality and execution of the study research designs using a written protocol to determine whether the study findings were at risk of bias. Studies were reviewed against standards that were similar to those from other review efforts, including the Teen Pregnancy Prevention Evidence Review (Goesling et al. 2014; U.S. Department of Health and Human Services 2017b), the Home Visiting Evidence of Effectiveness Review (U.S. Department of Health and Human Services 2017a), and the What Works Clearinghouse (2014). Reviewers assessed five potential components of bias: study design, confounding, reassignment, attrition, and baseline equivalence. The review process included regular meetings among the full review team to collectively address any differences of opinion or questions from reviewers. If any information was needed to assess risk of bias, reviewers queried studies’ authors. Authors were given three months to respond to questions; if they did not provide a response, the study was reviewed as it was publicly available.

#### Low Risk of Bias

We rated a study as having low risk of bias if it was an RCT with no confounds, such as systematic differences in the timing or method of data collection, and no reassignment of sample members across conditions. RCTs also had to have low attrition of sample members, defined using the What Works Clearinghouse conservative standards for overall and differential attrition (2014). If a study randomized clusters of participants to conditions (for example, couples or schools), it had to show low attrition at both the cluster and individual level. Cluster randomized trials with low attrition were considered at moderate risk of bias if sample members were added after random assignment. If an RCT with low attrition showed chance differences between groups on baseline demographics (age, race/ethnicity, or gender) or on a baseline measure of the outcome, it needed to control for that variable in analyses to be rated as at low risk of bias; if it did not, it was rated as at moderate risk of bias. A well-executed RCT is considered to provide evidence that is rigorous because randomization means that, on average, comparison groups will be similar on observable and unobservable characteristics.

#### Moderate Risk of Bias

If a study had high attrition, reassigned sample members to a different condition than the one to which they were originally assigned, or was a QED, we considered whether it was at moderate risk of bias. To be rated as moderate risk, a study had to demonstrate baseline equivalence for the analytic sample on (1) age, (2) race/ethnicity, (3) gender, and (4) a baseline measure of the outcome for which the study was testing impact. For studies that examined repeat pregnancy or birth, studies had to establish equivalence on a measure of baseline socioeconomic status (for example, income, employment, and welfare receipt) rather than a baseline measure of the outcome because establishing equivalence on being pregnant does not provide much information about the similarity of groups, given that all studies included expectant and parenting teens. Studies also had to control for baseline variables (or socioeconomic status) in analyses. Because these studies showed that comparison groups were similar, we consider them to provide rigorous evidence; however, we cannot be certain that the comparison groups did not differ on unobservable characteristics, so they are rated as having moderate (rather than low) risk of bias. For simplicity, throughout this article we refer to studies that are rated as at low or moderate risk of bias as rigorous.

#### High Risk of Bias

Studies were considered as having high risk of bias if they did not meet the criteria for low or moderate risk of bias. Because these studies could have had a confound or made comparisons across inequivalent groups, we do not have confidence in the evidence they provide.

#### Coding Risk of Bias Ratings for Outcome Domains

Studies often examined outcomes at more than one follow-up point, such as during the intervention and after the intervention ended. In addition, some studies evaluated outcomes in more than one domain. Risk of bias ratings could vary by follow-up and outcome domain if attrition varied across follow-ups or outcomes or because of different requirements for baseline equivalence across outcome domains. We categorized a study’s risk of bias for each outcome domain based on the follow-up with the lowest risk of bias within each domain.

### Data Extraction

#### Extraction of Study Outcomes

We extracted information about study outcomes, including the type of outcome measure, length of follow-up, and statistical significance of the impact estimate. For type of outcome measure, we coded whether outcomes fell into subcategories within outcome domains. For educational outcomes, we coded whether an outcome measured (1) educational progress, such as attendance or credit accumulation, or (2) educational attainment, such as receipt of a high school diploma or a General Educational Development (GED) certificate because attaining a credential may provide benefits for employment and income (Bjerk 2012; Campbell 2015; Oreopolous 2008). For repeat pregnancy or birth outcomes, we coded whether an outcome measured (1) a rapid repeat pregnancy or birth, defined as occurring within 24 months of the prior birth, or (2) other pregnancy or birth spacing outcomes, such as increasing the average length of time between births or reducing the total number of pregnancies or births teen parents experienced. Rapid repeat births may be particularly challenging to child and maternal health and well-being (Conde-Agudelo et al. 2006; Jones and Mondy 1994; Klerman 2004; Nerlander et al. 2015). We coded the length of follow-up as immediate if outcomes were measured during the program or fewer than two months after the end of the program; we coded the length of follow-up as longer-term if outcomes were measured more than two months after the end of the program because this indicates effects were maintained (or not) after the program ended.

When possible, we calculated effect sizes. For continuous outcomes, we calculated the standardized mean difference using Hedges’ *g*. For dichotomous outcomes, we calculated the difference in the probability of the occurrence of an event using the Cox index (What Works Clearinghouse 2014).

#### Categorizing Outcomes and Study and Program Effectiveness

First, we categorized each outcome; then we categorized study and program effects for each outcome domain.

##### Categorizing Outcomes

We categorized outcomes as favorable, unfavorable, or no effect, based on either evidence of (1) statistical significance (*p* < 0.05, based on a two-tailed hypothesis test) or (2) substantive effects based on an effect size of 0.25 or more. A favorable effect was a statistically significant or substantively important effect on an outcome for which the impact was in a direction that benefitted teen parents—for example, greater participation in education, more consistent contraceptive use, and fewer rapid repeat births. An unfavorable effect was a statistically significant or substantively important effect on an outcome for which the impact was in a direction that might harm teen parents—for example, increased drop out, not using contraception, and higher rates of repeat pregnancies. No effect was defined as when an effect was not statistically significant or substantively important.

##### Categorizing Effects for Studies

Although we documented effects from all follow-ups in all studies, we categorized a study as having favorable, unfavorable, or no effects for each outcome domain based on all of the rigorous follow-ups within each outcome domain. We considered a study to have favorable effects within a domain if there was at least one favorable effect and no unfavorable effects at any rigorous follow-up for that outcome domain. We considered a study to have unfavorable effects within a domain if there was at least one unfavorable effect at any rigorous follow-up for that outcome domain. We considered a study to have no effects within an outcome domain if there were no effects in all rigorous follow-ups for that outcome domain.

##### Categorizing Program Effectiveness

Some programs may have multiple studies of the programs’ effects. We considered programs effective if they had favorable effects (and no unfavorable effects) on outcomes in at least one of the domains of interest in at least one rigorous study.

#### Extraction of Study and Program Characteristics

For rigorous studies, we also extracted information about the study design and program characteristics. For study design, we examined the type of study; sample size; follow-up points; counterfactual comparison condition; and sample characteristics, including whether the sample included primarily African American participants, Latino participants, first-time parents, and low-income parents, based on whether more than three-quarters of the sample fell into a single category. We also noted whether the sample included fathers. For program characteristics, we examined the primary intervention strategy (for example, home visiting or case management); whether the program provided one-on-one support; the primary setting; program length; program frequency; and the type of facilitator. All program characteristics were based on the intended implementation described in the study.

## Results

In this section, we first describe the search and screening results. Next, we describe findings about study quality and what these findings suggest about potential biases in the reported effects. We then present the findings from rigorous studies for each outcome domain. We follow the PRISMA reporting checklist (Moher et al. 2009). We describe the outcomes of the studies through a qualitative synthesis rather than a quantitative meta-analysis because the studies’ designs, participants, programs, and reported outcome measures varied markedly. However, we present effect sizes for studies that included the necessary data to provide information about the magnitude and range of the effects. Finally, we discuss the characteristics of the programs evaluated in the studies considered rigorous for at least one outcome domain.

### Search and Screening Results

**Fig. 1:**
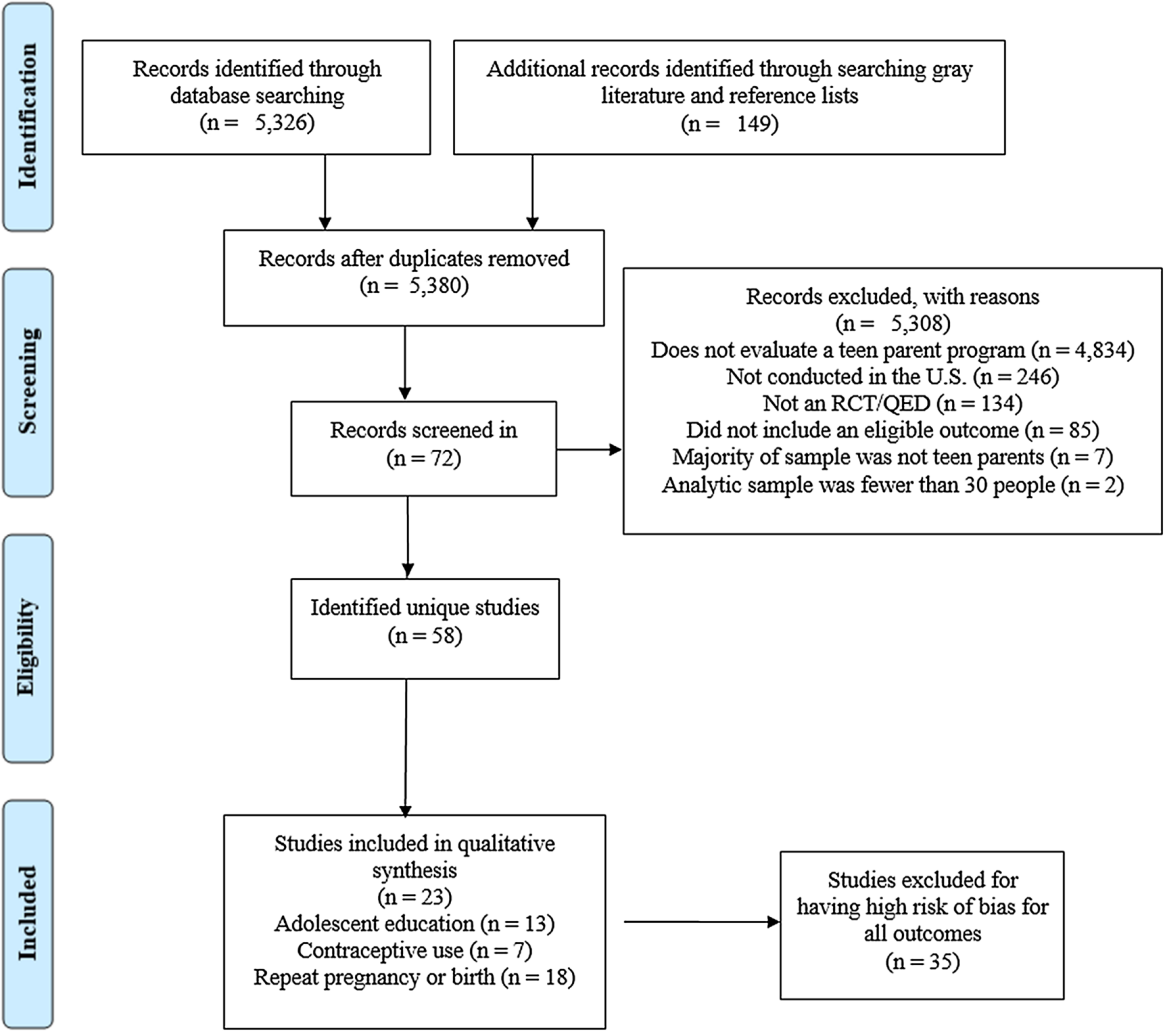
Search and screening results. *RCT* Randomized control trial, *QED *Quasi-experimental design

We identified a large number of unique potential records (n = 5380; Fig. 1). Most records were excluded because they did not evaluate a program for teen parents. After screening, 72 records were eligible, and these represented 58 unique studies, defined as a study of an eligible program with a unique sample.

### Findings About Study Quality

#### Study Risk of Bias

We categorized the number of studies as having favorable, no, or unfavorable effects within each outcome domain by whether the highest rated evidence for that domain is considered to be at low, moderate, or high risk of bias (Table 1). Within each outcome domain, most studies were considered to provide evidence that is at high risk of bias (59–65% of studies for each outcome domain).

**Table 1:** Percentage of studies with effects on teen parents’ education, contraceptive use, and repeat pregnancy or birth, by risk of bias

Risk of bias	Favorable effects n (%)	No effects n (%)	Unfavorable effects n (%)	Total with each quality rating n (%)
Teen parents’ education				
Low risk of bias	6 (19)	4 (13)	0 (0)	10 (31)
Moderate risk of bias	1 (3)	2 (6)	0 (0)	3 (9)
High risk of bias	18 (56)	1 (3)	0 (0)	19 (59)
Total	25 (78)	7 (22)	0 (0)	32 (100)
Contraceptive use				
Low risk of bias	2 (10)	2 (10)	0 (0)	4 (20)
Moderate risk of bias	1 (5)	1 (5)	1 (5)^a^	3 (15)
High risk of bias	10 (50)	3 (15)	0 (0)	13 (65)
Total	13 (65)	6 (30)	1 (5)	20 (100)
Repeat pregnancy or birth				
Low risk of bias	7 (16)	8 (18)	0 (0)	15 (34)
Moderate risk of bias	0 (0)	3 (7)	0 (0)	3 (7)
High risk of bias	17 (39)	9 (20)	0 (0)	26 (59)
Total	24 (55)	20 (45)	0 (0)	44 (100)
Highest study rating				
Low risk of bias	13 (22)	4 (7)	0 (0)	17 (29)
Moderate risk of bias	3 (5)	2 (3)	1 (2)^a^	6 (10)
High risk of bias	29 (50)	6 (10)	0 (0)	35 (60)
Total	45 (78)	12 (21)	1 (2)	58 (100)

Percentages may not sum to 100 because of rounding

^a^This study had favorable effects at one time point and unfavorable effects at another time point

#### The Proportion of Favorable Studies by Risk of Bias Rating

**Fig. 2:**
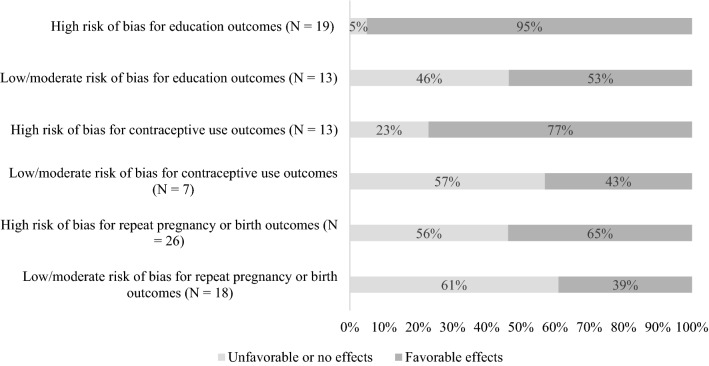
Proportion of studies with unfavorable/no effects versus favorable effects for each outcome domain, by risk of bias

As shown in Table 1, few studies had unfavorable effects, regardless of their study rating. Therefore, we consider studies with unfavorable/no effects together. Figure 2 shows the proportion of studies that had favorable versus unfavorable/no effects according to whether studies are rated as rigorous (at low or moderate risk of bias) or at high risk of bias for each outcome domain. For all outcome domains, studies rated as at high risk of bias were more likely to show favorable effects than studies that provided more rigorous evidence. This is particularly stark for studies of education outcomes. Nearly all studies (95%) rated as at high risk of bias showed favorable effects on an education outcome compared to slightly more than half (53%) of the studies rated as rigorous.

We excluded studies at high risk of bias in all outcome domains (N = 35; see Table 1) from the qualitative analysis of study characteristics and discussion of outcomes because we did not consider the evidence they provided to be rigorous. In addition, the findings illustrate that these studies demonstrated more favorable results than more rigorous studies did. This approach is similar to other review efforts that use study quality criteria to narrow the field of studies they discuss (Lachance et al. 2012; Klerman 2004). Therefore, the discussion of the study design characteristics and results for each outcome domain includes the 23 studies with rigorous evidence.

### Findings About Study Design and Sample Characteristics

Table 2 summarizes the study design and sample characteristics of the rigorous studies. Most studies were individual RCTs, with a small number of cluster RCTs. No QEDs met the criteria to be considered rigorous. Most studies only included one eligible follow-up considered rigorous. We calculated the mean and median sample size across studies by using the smallest rigorous follow-up for each study; these study follow-ups typically included 500–700 participants. More than half of studies compared the intervention to a usual care comparison group where comparison participants were not offered any services. Many of the studies that did not use a usual care comparison group compared the intervention to very light touch services such as information and referrals. Most studies included primarily first-time and low-income parents and were conducted in urban settings. Studies included a range of races/ethnicities, with some studies including primarily African American or Latino parents and other studies including participants from a range of racial/ethnic groups. Only four studies included fathers, and the proportion of fathers in each of these studies was less than 5%. Online Appendix Table A.3 provides details about the risk of bias ratings and sample characteristics for each rigorous study.

**Table 2:** Study design characteristics for rigorous studies

Characteristic	Number of studies or participants
Study design	
Individual RCT	20
Cluster RCT	3
Number of follow-ups that have an eligible outcome rated as rigorous	
One	14
Two	5
More than two	4
Follow-up sample sizes	
Smallest	73
Mean	717
Median	497
Largest	3498
Compared to usual care	13
Sample characteristics	
Primarily African American	6
Primarily Latino	4
Primarily first-time parents	17^a^
Primarily low-income	16^b^
Includes fathers	4
Setting	
Urban	17
Rural	2
Both	3

“Primarily” is used to designate that the evaluation sample included more than 75 percent of one group

^a^Two studies did not report whether parents were primarily first-time parents

^b^Four studies did not report whether parents were primarily low-income

### Findings About Effects on Aspects of Teen Parents’ Self-sufficiency

Table 3 summarizes the number of rigorous studies that improved outcomes in each domain. We then discuss findings for each outcome in turn: education, contraceptive use, and repeat pregnancy or birth.

**Table 3:** Number of studies with rigorous evidence with favorable or unfavorable/no effects

Outcomes	Favorable effects	Unfavorable/no effects	Total
Teen parents’ education	7	6	13
Educational progress	6	6	12
Educational attainment	2	7	9
Contraceptive use	3	4^a^	7
Repeat pregnancy or birth	7	11	18
Rapid repeat pregnancy or birth	6	3	9
Other repeat pregnancy or birth spacing outcomes	4	10	14
Total	16	7	23

^a^One study counted as having unfavorable/no effects had favorable effects at one time point and unfavorable effects at another time point

#### Education

**Fig. 3:**
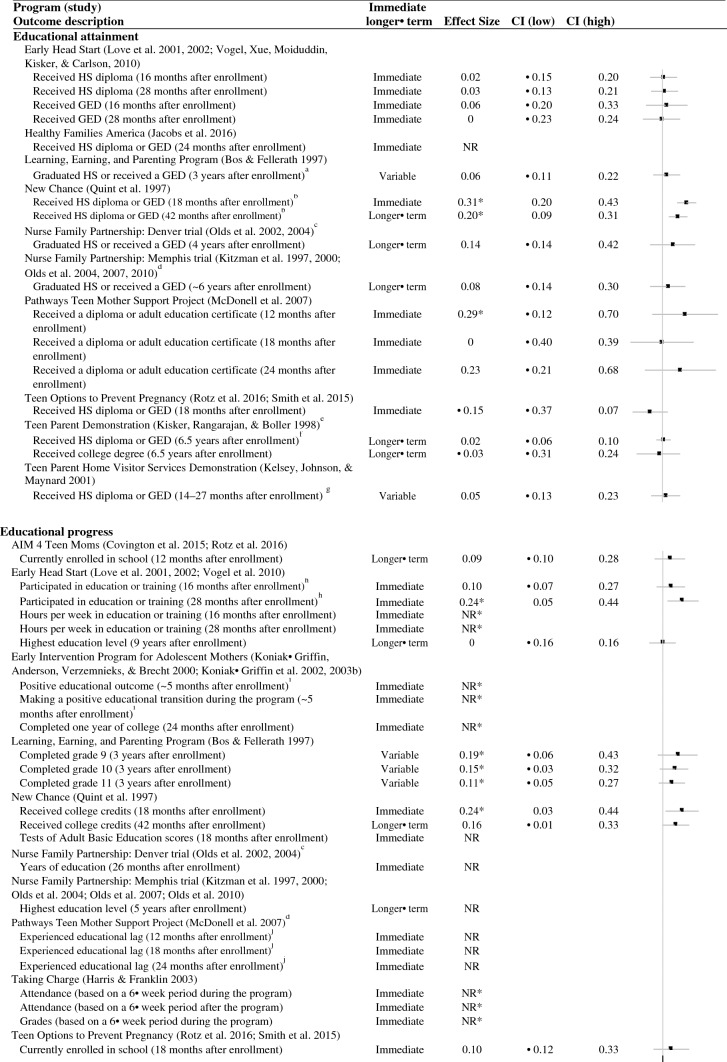

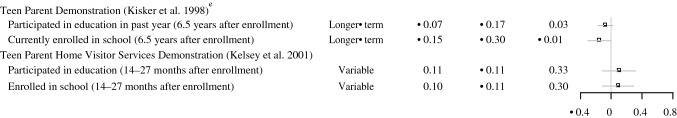
Effect sizes for educational outcomes. *Indicates the effect was statistically significant or substantively important. *NR *effect size not reported, *CI *confidence interval, *HS* high school. ^a^When the effects of Learning, Earning, and Parenting Program on graduating high school or receiving a GED are separated, there are no effects on either outcome. ^b^When the effects of New Chance on receiving a high school diploma or receiving a GED are separated, there are favorable effects on receiving a GED and unfavorable effects on receiving a high school diploma. ^c^For the Denver Nurse Family Partnership trial, we focused on comparing the nurse home visiting group with the developmental screening comparison group. Unlike the favorable effects found for the nurse home visiting group, the study found no effects on any pregnancy/birth outcomes for the paraprofessional home visiting group. ^d^We calculated all results for the study of Pathways Teen Mother Support Project (based on an author query because the authors only provided results for a combined follow-up that would have received a low rating). ^e^We combined effects across the three study sites in the Teen Parent Demonstration because we considered this a single study according to the What Works Clearinghouse Procedures Handbook Version 4 (2017). We could do this only for particular outcomes that provided the necessary information (for example, high school diploma or GED receipt, but not highest grade completed). ^f^When the effects of Teen Parent Demonstration on receiving a high school diploma or receiving a GED are separated, there are no effects on either outcome. ^g^When the effects of Teen Parent Home Visitor Services Demonstration on receiving a high school diploma or receiving a GED are separated, there are no effects on either outcome. ^h^The study of Early Head Start also looked at whether participants were ever in high school. Results were consistent with whether participants participated in any education or training, except those in the treatment group were significantly more like to have been in high school at the 16-month follow-up. ^i^A positive educational outcome and transition for Early Intervention Program for Adolescent Mothers was defined as attending high school or junior college or graduating from high school versus being enrolled in school and not attending or dropping out of school. ^j^Here a negative coefficient would represent a favorable effect. ^k^There were positive effects on educational progress in an earlier follow-up of the Teen Parent Demonstration that was published before the review eligibility period

As shown in Table 3, slightly more than half of the studies that rigorously evaluated an educational outcome showed at least one favorable effect (7 of 13 studies); six studies did not show any favorable effects. Most studies measured and showed favorable effects on education during the program or immediately after the program (six of nine studies; Fig. 3). Only one study that measured outcomes more than two months after the end of the program showed any longer-term improvements in teen parents’ educational outcomes (one of seven studies; Fig. 3).

Studies were more likely to improve teens’ educational progress (such as attendance or credit accumulation) than their educational attainment (such as receipt of a high school diploma or GED completion). Specifically, half of the studies that measured educational progress showed at least one favorable effect on progress (6 of 12 studies). In contrast, fewer than one-quarter of studies that measured attainment showed at least one favorable effect on attainment (two of nine studies). In addition, the two studies that showed at least one favorable effect on attainment had somewhat mixed effects. New Chance showed favorable effects on a combined measure of either GED or high school diploma receipt. However, these effects were driven by favorable effects on GED receipt; there were unfavorable effects on receiving a high school diploma (Quint et al. 1997). Pathways Teen Mother Support Project showed favorable effects on receiving a diploma or adult education certificate at one follow-up point but did not show effects at two other follow-up points (McDonell et al. 2007).

Across all 13 rigorous studies of education, there were 42 rigorous comparisons of educational progress or attainment. We could calculate effect sizes for 27 comparisons (Fig. 3). For educational attainment, we could calculate 16 effect sizes from nine of the ten studies that examined attainment; effects ranged from − 0.15 to 0.31. Although few effects were statistically significant or substantial, most were slightly favorable (14 of 16 effect sizes). For educational progress, we could calculate 14 effect sizes from 7 of the 12 studies that examined progress; effects ranged from − 0.21 to 0.25. Again, most effects were slightly favorable (12 of 14 effects).

#### Contraceptive Use

**Fig. 4:**
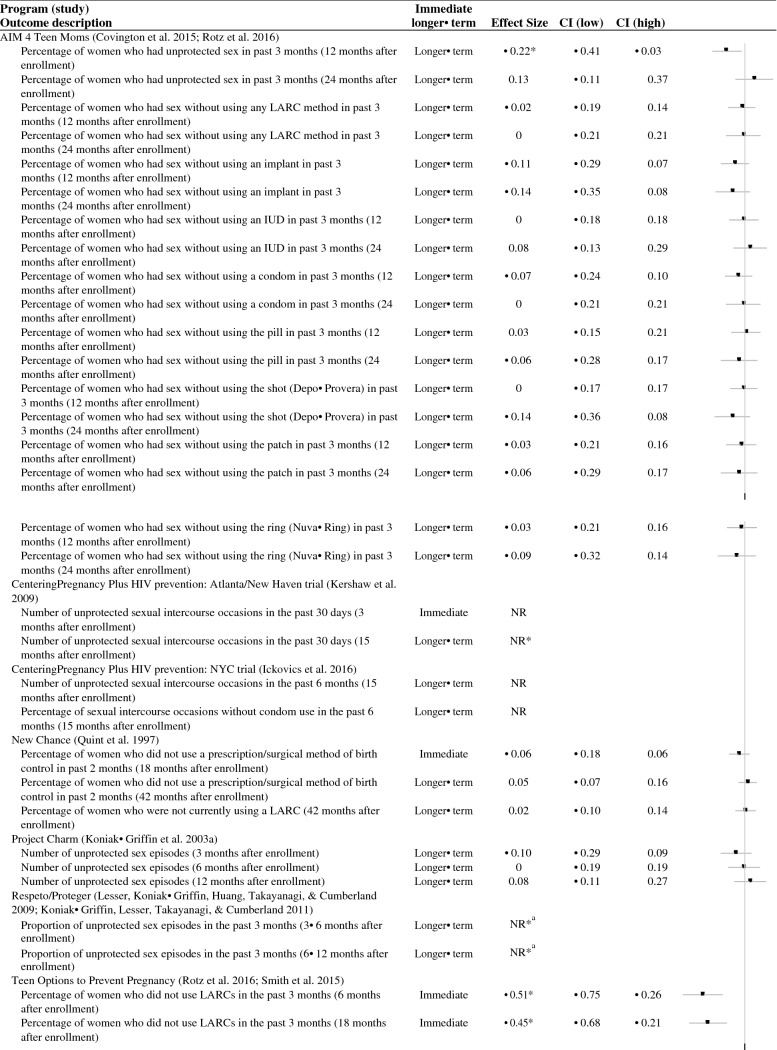

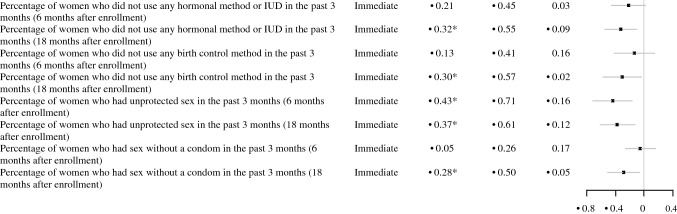
Effect sizes for contraceptive use outcomes. *Indicates the effect was statistically significant or substantively important. *NR* effect size not reported, *CI* confidence interval, *LARC *long acting reversible contraceptive, *IUD* intrauterine device. ^a^Immediate impacts of Respeto/Proteger were favorable (reductions in unprotected sexual episodes), but longer-term impacts were unfavorable (increases in unprotected sexual episodes)

In total, slightly fewer than half of the studies that examined contraceptive use demonstrated favorable effects on a measure of contraceptive use (three of seven studies; Table 3). One study (of three studies) showed favorable effects on contraceptive use during or immediately after the end of the program, and two studies (of six studies) showed favorable effects on contraceptive use more than two months after the program ended (Fig. 4). In addition, one study showed a favorable effect in reducing unprotected sex in an earlier follow-up and then an unfavorable effect in increasing unprotected sex in a later follow-up.

Across the seven studies with rigorous evidence, there were 40 rigorous comparisons of contraceptive use. We could calculate effect sizes for 34 comparisons from four studies (Fig. 4). Although most effects were not statistically significantly different from zero, most effects were slightly favorable (23 of 33 effects).

#### Repeat Pregnancy or Birth

In total, more than one-third of the studies that examined repeat pregnancy or birth showed favorable effects on at least one repeat pregnancy or birth outcome (7 of 18 studies; Table 3). Some studies measured rapid repeat pregnancy or birth, defined as occurring within 24 months of the prior birth;^1^ some studies measured other repeat pregnancy or birth spacing outcomes, such as the total number of births. Studies that measured rapid repeat pregnancy were more likely to show favorable effects (6 of 9 studies) than studies that measured other pregnancy or birth spacing outcomes (4 of 14 studies).

**Fig. 5:**
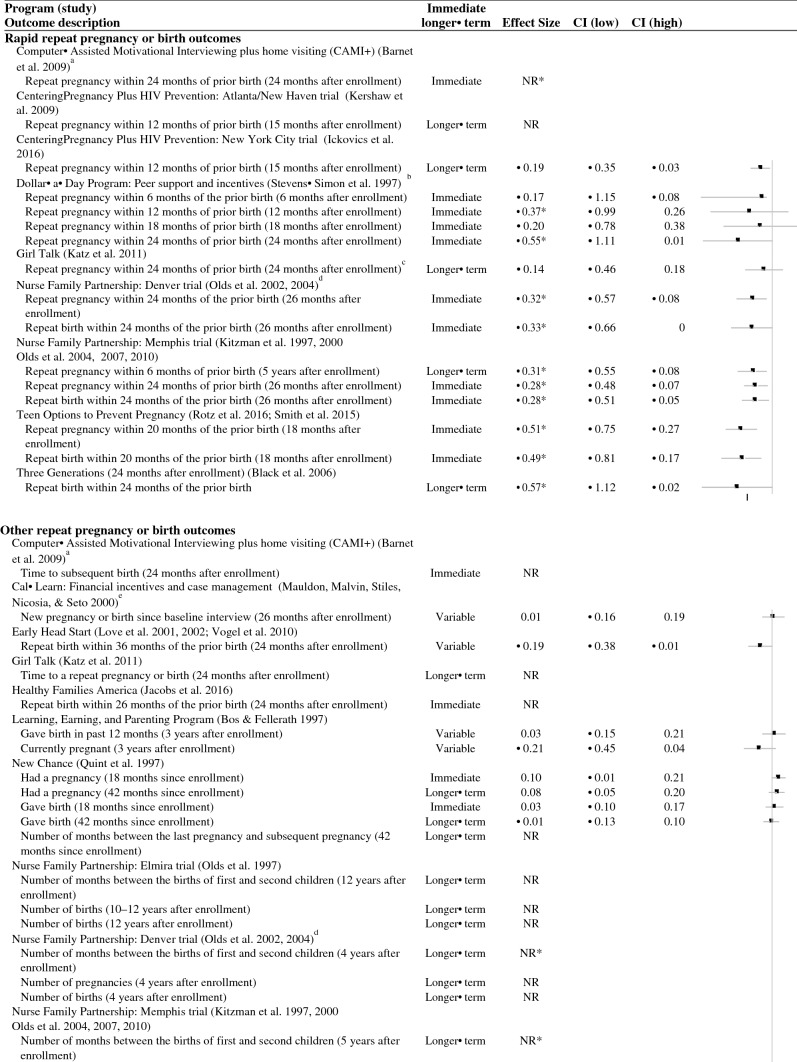

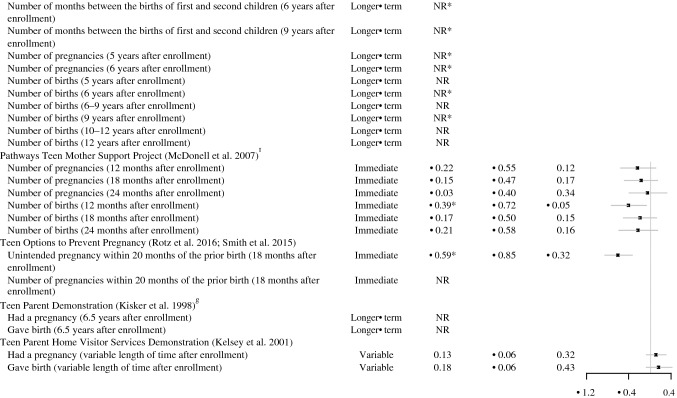
Effect sizes for repeat pregnancy or birth outcomes. *Indicates the effect was statistically significant or substantively important. *NR* effect size not reported, *CI* confidence interval, *CAMI* computer-assisted motivational interviewing. ^a^The comparison of a CAMI-only group with a usual care group was also considered rigorous, but we focus on reporting results for the CAMI plus home visiting condition versus usual care comparison. There were no effects on repeat birth or time to subsequent birth for a CAMI-only group compared with a usual care group. ^b^In the Dollar-a-Day program, the comparison of the incentive and peer support group to an incentive-only group was also considered rigorous. However, we focus on the comparison of the incentive and peer support group with the peer support-only group because only two participants took part in the peer support-only group, so it is similar to a no-treatment comparison group. Participants in the incentive and peer support group had lower rates of repeat pregnancy than the incentive-only group within 6 months of the prior birth during the program (6 months after enrollment), but there were no effects on repeat pregnancy within 12, 18, and 24 months of the prior birth during the program (12, 18, and 24 months after enrollment). ^c^We calculated effects for having a repeat pregnancy for the Girl Talk intervention, but the study only analyzed time to a repeat pregnancy. ^d^For the Denver Nurse Family Partnership trial, we focus on comparing the nurse home visiting group with the developmental screening comparison group. Unlike the favorable effects found for the nurse home visiting group, the study found no effects on any pregnancy/birth outcomes for the paraprofessional home visiting group compared to developmental screening. ^e^Cal-Learn had four comparison groups. We present findings for the full Cal-Learn intervention (financial incentive and case management) to the usual treatment comparison. ^f^We calculated all results for the study of Pathways Teen Mother Support Project based on an author query because the authors only provided results for a combined follow-up that would have received a low rating. ^g^We combined effects across the three study sites in the Teen Parent Demonstration because we considered this a single study according to the What Works Clearinghouse Procedures Handbook Version 4 (2017). We could only do this for particular outcomes that provided the necessary information (for example, high school diploma or GED receipt, but not highest grade completed)

Across all 18 studies of repeat pregnancy or birth, there were 56 rigorous comparisons of a repeat pregnancy or birth outcome (Fig. 5). We could calculate effect sizes for about half of these comparisons (30 effect sizes from 14 studies). For rapid repeat pregnancy, all of the 14 effect sizes (from seven of the nine studies of rapid repeat pregnancy) showed that the treatment group had lower rates of rapid repeat pregnancy than did the comparison group, but these differences were not always statistically significant. Effect sizes ranged from − 0.57 to − 0.14. For other repeat pregnancy or birth outcomes, the findings were less consistent. We could calculate 16 effects from seven studies. Ten of these effects were slightly favorable, and six were slightly unfavorable; effects ranged from − 0.59 to 0.13.

#### Findings Across Outcomes

In total, ten studies had rigorous comparisons in two domains and two studies had rigorous comparisons in all three domains. Of the 12 studies that rigorously examined more than one domain, two studies showed favorable effects in more than one domain. Pathways Teen Mother Support Project showed favorable effects on education and repeat pregnancy or birth (McDonell et al. 2007). Teen Options to Prevent Pregnancy showed favorable effects on contraceptive use and repeat pregnancy or birth (Smith et al. 2015; Rotz et al. 2016).

#### Overall Study and Program Findings

Overall, 17 (of 23) studies showed at least one favorable effect on one of the target outcomes. These 23 studies represent 20 programs because two programs had more than one rigorous study of their program. One of the studies of CenteringPregnancy Plus showed favorable effects on contraceptive use (Kershaw et al. 2009), whereas the other did not (Ickovics et al. 2016). Two of the three studies of Nurse Family Partnership that measured repeat pregnancy or birth showed favorable effects (Olds et al. 2002, 2004, 2007, 2010; Kitzman et al. 1997, 2000). Neither of the Nurse Family Partnership studies that examined educational outcomes showed effects (Olds et al. 2002, 2004, 2007, 2010; Kitzman et al. 1997, 2000). Therefore, 14 (of 20) programs showed at least one favorable impact on one of the target outcomes. Few programs have been studied rigorously more than once; as such, there is limited replication of favorable findings across more than one study of a program. We discuss the characteristics of the 20 rigorously examined programs next.

### Program Characteristics

The 20 programs that rigorously evaluated an eligible self-sufficiency outcome had diverse characteristics (Table 4; see Online Appendix Table A.4 for program descriptions). Given the small number of programs with rigorous evidence, we cannot determine whether any program features are associated with effectiveness, but we describe the characteristics of the 14 programs that improved at least one of the target outcomes. Effective programs were implemented in many different settings and used diverse primary intervention strategies, although more than half used either home visiting (six programs) or case management (three programs). Most effective programs provided one-on-one support to teens, either on their own (six programs) or alongside other strategies such as group meetings (five programs). Slightly more than half of programs were implemented by case workers who do not have a professional license (eight programs); others were implemented by teachers, social workers, nurses, and clinicians. Finally, many effective programs were intensive—they provided at least biweekly contact (nine programs) for longer than a year (nine programs). However, programs that did not improve one of the target outcomes shared many of these characteristics. Some programs that improved educational outcomes primarily focused on improving academic outcomes, whereas others focused on improving a range of outcomes. In contrast, most of the programs that improved outcomes related to healthy birth spacing (contraceptive use or repeat pregnancies or births) identified supporting healthy birth spacing as their primary goal.

**Table 4:** Characteristics of programs that improved (or did not improve) teen parents’ self-sufficiency outcomes

Characteristic	Number of programs that improved outcomes	Number of programs that did not improve outcomes
Primary intervention strategy		
Case management	3	2
Comprehensive family support services	1^a^	0
Educational or employment services	1	1
Financial incentive	1	0
Group-based curriculum	1	2
Home visiting	6	1
Prenatal care	1	0
Mode of intervention		
One-on-one plus other strategies^b^	5	1
One-on-one	6	3
Small group	3	1
Couple-focused	0	1
Type of facilitator		
Case worker^c^	8	4
Clinician	1	0
Teacher	1^a^	0
Nurse	3	1
Social worker	1	1
Primary setting		
Community based	3	1
Clinic	1	0
Home	6	1
School	2^a^	1
Telephone	1	1
Welfare Office	1	2
Length		
Less than 3 months	2	2
3 to 12 months	3	0
More than 12 months	9	3
Variable length	0	1
Frequency		
Daily	1	0
At least weekly	5	2
At least biweekly	3	3
At least monthly	3	0
Variable frequency	2	1
Total	14	6

^a^The study of Early Head Start included the early childhood education (ECE) based, daily intervention and the home visiting intervention. The characteristics in the table reflect the ECE-based version

^b^Programs that provide one-on-one support plus other strategies use a number of different approaches. For example, a program may primarily offer one-on-one home visiting but also offer small-group parent training sessions

^c^A case worker does not have a professional license. Some programs require case workers to have a certain educational qualification, but others do not

## Discussion

In this systematic review of the evidence about programs for expectant and parenting teens, we found rigorous evidence that varied types of programs can support aspects of teens’ self-sufficiency by improving outcomes related to education and healthy birth spacing. Overall, 23 studies were considered to provide rigorous evidence about either education, contraceptive use, or repeat pregnancy or birth; 17 of these studies showed at least one favorable effect in at least one of these domains and 6 did not show any significant or substantial effects in these domains. In total, 14 programs improved outcomes related to aspects of teen parents’ self-sufficiency, which can inform practitioners seeking to promote teens’ self-sufficiency. Here, we first discuss findings about study quality, next discuss findings about each outcome domain, and then describe the characteristics of effective programs. Finally, we discuss the limitations of this review. Throughout, we draw attention to implications for policy and program development and the need for future research.

### State of the Evidence

Although we identified some rigorous studies that came from well-conducted RCTs or QEDs that showed that comparison groups were similar, fewer than half of the eligible studies provided evidence considered rigorous; this is consistent with other reviews (U.S. Department of Health and Human Services 2017a, b). Moreover, in all three domains, evidence considered at high risk of bias was substantially more likely to show favorable effects than evidence considered rigorous. This finding is consistent with prior research (Steinka-Fry et al. 2013; Whitaker et al. 2016). For example, a meta-analysis of programs to increase educational attainment for low-income African American teen mothers found that quasi-experimental studies were more likely to show favorable effects than were randomized studies (Baytop 2006). This demonstrates the importance of conducting this type of review so that practitioners have access to information about programs that work based on rigorous evidence. However, rigorous evidence is particularly lacking about some outcomes and populations. For example, we found only a handful of rigorous studies of contraceptive use for teen parents, despite the demonstrated importance of contraceptive use in preventing rapid repeat teen pregnancies (Bennett et al. 2006; Coard et al. 2000; Raneri and Wiemann 2007; Stevens-Simon et al. 2001). In addition, only four rigorous studies included teen fathers, and teen fathers only made up a small percentage of participants in these studies. Moreover, more research is needed to determine whether effects can be replicated with different populations and with greater scale because most programs only have evidence of effectiveness from one rigorous study. Nonetheless, this body of evidence also has strengths: most of the rigorous evidence that exists was from studies with randomized designs and moderately large samples that provide insight into how to support vulnerable teen mothers from multiple ethnic/racial groups.

### Understanding Effects on Education, Contraceptive Use, and Repeat Pregnancy

Increasing education and contraceptive use and reducing repeat pregnancies or births are central aspects of promoting teen parents’ self-sufficiency (Assini-Meytin and Green 2015; Bjerk 2012; Campbell 2015; Diaz and Field 2016; Jones and Mondy 1994; Klerman 2004; Lee 2010; Oreopolous 2008). We discuss findings for each outcome domain in turn. About half of the 13 studies that rigorously examined an educational outcome showed favorable effects. The evidence that programs can promote teens’ educational outcomes is consistent with an earlier meta-analytic review of programs to reduce dropout that found favorable effects of programs for teen parents on their graduation and enrollment (Steinka-Fry et al. 2013). In addition, these findings are consistent with findings from an evaluation of the New Heights program that used an interrupted time series design with multiple comparison groups, presented in this supplement (Zief et al., forthcoming). This study of New Heights addressed sources of bias to provide credible evidence of favorable effects on teen mothers’ educational progress, with suggestive evidence of favorable effects on teen mothers’ educational attainment. Similar to the findings from New Heights, studies were more likely to show favorable effects on educational progress, such as school enrollment, than on educational attainment, such as receipt of a GED or high school diploma. This may be because short follow-up periods do not provide enough time to observe impacts on attainment or could suggest that gains in measures of progress do not always translate to gains in attainment. To stay in school for long enough to receive a credential, teens may require financial support, given the substantial challenges they face in supporting themselves and their children (Acs and Koball 2003; Assini-Meytin and Green 2015; Diaz and Field 2016; Lee 2010). Although credential attainment might be important for job opportunities and future income (Bjerk 2012; Campbell 2015; Oreopolous 2008), educational progress may still increase parents’ skills and benefit them and their children. For example, maternal participation in education has been associated with improvements in parenting (Harding et al. 2017).

Nearly half of the seven rigorous studies of contraceptive use demonstrated favorable effects on at least one outcome. Some of these favorable effects were sustained more than nine months after the program ended. These findings are consistent with recent evidence from a study of a home visiting program, Steps to Success, that was released after the eligibility period for the current review; this evidence supports that programs can increase teen mothers use of the most effective methods of birth control, including long acting reversible contraceptives (Rotz and Wood 2018). As far as we are aware, this systematic review is the first to examine whether programs can impact teen parents’ contraceptive use, which is an important way to support healthy birth spacing (Bennett et al. 2006; Raneri and Wiemann 2007; Stevens-Simon et al. 2001; Coard et al. 2000).

More than one-third of the 18 studies that rigorously examined an outcome related to repeat pregnancy or birth showed favorable effects. Evidence that programs can reduce repeat births is consistent with a prior meta-analysis that showed a 50% reduction in the odds of pregnancy around 19 months after enrollment in the study (Corcoran and Pillai 2007). Studies that measured rapid repeat pregnancy or birth, defined as occurring within 24 months of the prior birth, were more likely to show effects than studies that measured other repeat pregnancy or birth spacing outcomes, such as the total number of births. Programs may be more likely to delay repeat pregnancy rather than reduce the number of children parents have overall. Given the risks of rapid repeat birth for pre-term or still births (Conde-Agudelo et al. 2006; Nerlander et al. 2015), these programs may contribute to important maternal and child health outcomes. None of the studies that improved outcomes related to healthy birth spacing (contraceptive use or repeat pregnancies or births) included fathers, so we know very little about the effectiveness of programs in increasing fathers’ contraceptive use and reducing their rates of contributing to rapid repeat births.

Overall, there is evidence that programs can promote outcomes related to teen parents’ self-sufficiency, although effects were typically small. For each outcome domain, about one-third to one-half of the rigorous studies showed at least one favorable effect, with 17 studies demonstrating improved outcomes. The six studies that did not demonstrate any effects on education or healthy birth spacing may have favorably impacted other outcomes for teen parents. This proportion of favorable studies is higher than the proportion of favorable programs from the Teen Pregnancy Prevention Evidence Review in which 31 of 78 programs showed favorable effects on at least one outcome of interest (Goesling et al. 2014).^2^ Notably, most studies of programs for teen parents showed favorable effects only on one domain of interest, which may indicate programs are better at improving specific outcomes than outcomes across both education and healthy birth spacing. In addition to some studies examining outcomes in more than one domain, many studies examined more than one outcome within domains. For the outcomes for which we could calculate effect sizes, most effects suggested slightly favorable outcomes for teen parents, even when they were not statistically or substantially different from zero. Few effects were unfavorable, suggesting that programs do not harm teen parents.

### Characteristics of Programs to Support Expectant and Parenting Teens

Programs to support expectant and parenting teens have diverse characteristics, indicating there is no single approach for promoting teens’ education and healthy birth spacing (Corcoran and Pillai 2007; Steinka-Fry et al. 2013). For example, Teen Options to Prevent Pregnancy provided telephone-based motivational interviewing intervention to promote healthy birth spacing (Smith et al. 2015; Rotz et al. 2016), CenteringPregnancy Plus provided group-based prenatal care and education about healthy birth spacing (Kershaw et al. 2009; Ickovics et al. 2016), and New Chance provided educational and employment services to welfare-eligible teens through community organizations (Quint et al. 1997). Effective programs used many different primary intervention strategies, with many using home visiting. Home visiting programs have been found to improve other parent and child outcomes, so they may be one promising intervention strategy to support teen parents (Sama-Miller et al. 2018). Even among programs that used similar strategies, programs had different goals. Programs that reduced rapid repeat births tended to focus on this as a primary outcome, but some effective programs focused on improving parent and child outcomes broadly. In general, effective programs typically provided intensive one-on-one support. However, programs that did not improve one of the target outcomes had characteristics similar to programs that did improve a target outcome, so more research is needed to understand how program features and implementation contribute to program effectiveness.

### Limitations

Although this systematic review provides a comprehensive analysis of nearly 60 studies of programs to support expectant and parenting teens, there are some limitations. First, we used study eligibility and rating criteria that can result in the exclusion of evidence that may be credible, such as the study of New Heights featured in this supplement (Zief et al., forthcoming). Although we used criteria similar to those used by other systematic reviews and such criteria are useful for ensuring consistency, some experts have criticized using such criteria to categorize studies (Jüni et al. 1999). Nonetheless, we considered categorizing some studies as having high risk of bias to be helpful in narrowing the field of studies to discuss, given these studies showed substantially more favorable effects than did more rigorous studies. Second, although we included both published and unpublished literature to address potential publication bias, researchers may have additional relevant findings that are currently unavailable in any public written report. Third, we focused on two key aspects of self-sufficiency—teen parents’ education and healthy birth spacing. However, there are other important aspects of self-sufficiency, such as stable employment, and other factors that can support self-sufficiency, such as mental well-being. Studies that did not show favorable impacts on the outcomes eligible for this systematic review could have impacted other important outcomes. Fourth, we used a wide date range to identify a number of relevant studies. However, the policy context and characteristics of teen parents have changed (Driscoll 2014), which could limit generalizability to the present day. Finally, many studies did not present the information needed to calculate effect sizes, and we did not identify a large enough number of rigorous studies to conduct a meta-analysis or distinguish effective program features. In addition, we cannot provide information about the cost of implementing the effective programs because studies of costs are typically limited. Therefore, although we identified 14 programs that improve aspects of teen parents’ self-sufficiency, we echo prior calls for more rigorous studies of programs to support teen parents in achieving self-sufficiency.

## Electronic supplementary material

Below is the link to the electronic supplementary material.

Supplementary file1 (DOCX 67 kb)

## Footnotes

1 Note that some studies included as measuring rapid repeat pregnancy or birth measured these outcomes less than 24 months since the prior pregnancy or birth.

2 These 78 programs represent 88 studies, but information about the number of studies with favorable effects was not available.
