# Supplementary material for: A Systematic Review of Programs to Promote Aspects of Teen Parents’ Self-sufficiency: Supporting Educational Outcomes and Healthy Birth Spacing
Source: Matern Child Health J. 2020 Jan 21;24(Suppl 2):84–104. doi: 10.1007/s10995-019-02854-w (PMC7497377; doi:10.1007/s10995-019-02854-w)
Supplement: Supplementary file 1 — Supplementary file1 (DOCX 67 kb) [file 10995_2019_2854_MOESM1_ESM.docx]

Appendix Table A.1

*Study search details*

| Search characteristics | Limits |
| --- | --- |
| Dates | After December 31, 1996, until March 2017 |
| Language | English^a^ |
| Age | Adolescent (13 to 18 years) |
| Age | Young adult (19 to 24 years) |
| Fields | Title, abstract, medical subject heading (MeSH) |
| Duplicates | Duplicates are removed from the search and from the result count |
| Databases | MEDLINE, Scopus, CINAHL, PsychINFO, ERIC |

^a^ Studies conducted in the United States can be reported in journals located in other countries or by authors affiliated with academic institutions outside of the United States.

Appendix Table A.2

*Study search terms*

| Set | Concept | Keywords (title, abstract, keyword fields) | Subject terms (MeSH) | |
| --- | --- | --- | --- | --- |
| 1 | Population | "adolescen* parent*" OR "adolescen* father*" OR "adolescen* mother*" OR "adolescen* step*" OR "emerging adult* parent*" OR "emerging adult* father*" OR "emerging adult* mother" OR "emerging adult* step*" OR "juvenile* parent*" OR "juvenile* father*" OR "juvenile* mother*" OR juvenile* step*" OR "teen* parent*" OR "teen* father*" OR "teen* mother*" OR "teen* step*" OR "young adult* parent*" OR "young adult* father*" OR "young adult* mother*" OR "young adult* step*" OR "young parent*" OR "young father*" OR "young mother*" OR "young step*" OR adolescen* n/3 pregnanc* OR emerging adult* n/3 pregnanc* OR juvenile* n/3 pregnanc* teen* n/3 pregnanc* OR "young adult" n/3 pregnanc* OR "young people" n/3 pregnanc* OR "youth*" n/3 pregnanc* | (adolescent[MeSH] OR young adult[MeSH]) AND "pregnancy"[MeSH] | |
| 2 | Study design | (study OR evaluat* OR research) AND (effective* OR efficac* OR impact* OR outcome* OR implement* OR cost* OR replic*) |  | |
| 3 | Study design | QED OR "quasi experiment" OR quasiexperiment* OR "randomize* control* trial*" OR RCT OR "comparison group*" | "Randomized Controlled Trials as Topic"[MeSH] | |
| 4 | 2 OR 3 |  |  | |
| 5 | 1 AND 4 | *Notes: Studies of expectant and parenting teens and young adults* |  | |
| 6 | Outcome: contraception OR condom | contracep* OR "birth control" OR "pregnancy prevention" OR condom* | “Contraception”[ MeSH] OR "Condoms"[ MeSH] | |
| 7 | 5 AND 6 | *Notes: Studies of expectant and parenting teens and young adults using contraception or condoms* |  | |
| 8 | Outcome: repeat pregnancy or birth | repeat OR multiple OR subsequent OR gravid OR multigravida OR secundigravida |  | |
| 9 | 5 AND 8 | *Notes: Studies of expectant and parenting teens and young adults with repeat pregnancies* |  | |
| 10 | Outcome: Academics | (graduat* OR "academic success" OR "drop out" OR dropout OR promotion* OR matriculat* OR "academic achievement*" OR "high school diploma*" OR "high school degree*" OR attendance OR GPA OR "standardized achievement") |  | |
| 11 | 5 AND 10 | *Notes: Studies of expectant and parenting teens and young adults and their educational outcomes* |  | |
| 12 |  | ("home visit*" OR "home-visit*") |  | |
| 13 | 5 AND 12 | *Notes: Studies of expectant and parenting teens and young adults and home visiting* |  | |
| 14 | Combine sets | 7 OR 9 OR 11 OR 13 |  | |
| 15 | Editorial | letter* OR "newspaper article*" OR commentar* OR editorial* OR opinion* OR "op-ed*" OR conference* |  | |
| 16 | Combine sets | 14 NOT 15 |  |  |

Note: MeSH = medical subject heading.

Appendix Table A.3

*Study details for rigorous studies*

| **Program (citation)** | | |  |  |
| --- | --- | --- | --- | --- |
| **Education** | **Contracept-ive use** | **Repeat pregnancy or birth** | **Study type and explanation of rating** | **Study sample and setting** |
| AIM 4 Teen Moms (Covington et al. 2015; Rotz et al. 2016) compared to usual care | | | | |
| Moderate risk | Moderate risk | High risk | Individual RCT with high attrition that established equivalence on education and contraceptive use but did not establish equivalence on SES to meet requirements for repeat pregnancy or birth (two rigorous follow-ups; N = 529–800) | Primarily Latino, first-time, low-income mothers in an urban setting (Los Angele, CA)^a^ |
| Cal-Learn: Financial incentives and case management (Mauldon et al. 2000) compared to usual care | | | | |
| High risk | High risk | Moderate risk | Individual RCT with high attrition at one follow-up that established equivalence on SES to meet requirements for repeat pregnancy or birth but did not establish equivalence on education or contraceptive use (N = 2,023) | Primarily first-time, low-income mothers in rural and urban settings (Alameda, CA; Los Angeles, CA; San Bernardino, CA; San Joaquin, CA) |
| CenteringPregnancy Plus HIV prevention: Atlanta/New Haven trial (Kershaw et al. 2009) compared to usual prenatal care | | | | |
|  | Low risk | Low risk | Individual RCT with low attrition (two rigorous follow-ups; N = 840–934) | Primarily African American mothers in urban settings (New Haven, CT; Atlanta, GA)^b^ |
| CenteringPregnancy Plus HIV prevention: New York City trial (Ickovics et al. 2016) compared to usual prenatal care | | | | |
|  | Moderate | Moderate | Cluster RCT in 14 clinical sites. Recruitment occurred after random assignment (N = 1148) | Primarily first-time, low-income mothers in an urban setting (New York, NY)^c^ |
|  |  |  |  |  |
| **Education** | **Contracept-ive use** | **Repeat pregnancy or birth** | **Study type and explanation of rating** | **Study sample and setting** |
| Computer-Assisted Motivational Intervention plus home visiting (Barnet et al. 2009) compared to usual care | | | | |
|  |  | Low risk | Individual RCT with low attrition that controlled for chance differences between the treatment and comparison groups^d^ (N = 148) | Primarily African American, first-time, low-income mothers in an urban setting (Baltimore, MD) |
| Dollar-a-Day Program: Peer support and incentives (Stevens-Simon et al. 1997) compared to peer support only | | | | |
|  |  | Low risk | Individual RCT with low attrition for the comparison to the peer support-only group^e^ (N = 120) | Primarily first-time, low-income mothers in an urban setting (Denver, CO) |
| Early Head Start (Love et al. 2001, 2002; Vogel et al. 2010) compared to usual care | | | | |
| Low risk |  | Low risk | Individual RCT with low attrition for the first two rigorous follow-ups (N = 788–854); high attrition for the third rigorous follow-up that showed baseline equivalence for education (N = 653)^f^ | Primarily low-income mothers and fathers (1 percent) in urban and rural settings (17 programs across the U.S., half urban, half rural) |
| Early Intervention Program for Adolescent Mothers (Koniak-Griffin et al. 2000, 2002, 2003b) compared to traditional public health nursing (three home visits) | | | | |
| Low risk |  |  | Individual RCT with low attrition (N = 121) | Primarily first-time, low-income mothers in an urban setting (San Bernardino, CA) |
| Girl Talk (Katz et al. 2011) compared to usual care | | | |  |
|  |  | Low risk | Individual RCT with low attrition (N = 237) | Primarily African American, first-time mothers in an urban setting (Washington, DC) |
| Healthy Families America (Jacobs et al. 2016) compared to usual care and information/referrals | | | | |
| Low risk |  | Low risk | Individual RCT with low attrition (N = 612) | Primarily first-time mothers (18 sites across MA)^g^ |
| **Education** | **Contracept-ive use** | **Repeat pregnancy or birth** | **Study type and explanation of rating** | **Study sample and setting** |
| Learning, Earning, and Parenting Program (Bos & Fellerath 1997) compared to usual care | | | |  |
| Low risk |  | Low risk | Individual RCT with low attrition (N = 968) | Primarily first-time, low-income mothers and fathers (2 percent) in urban and rural settings (Cincinnati, OH; Cleveland, OH; Toledo, OH) |
| New Chance (Quint et al. 1997) compared to usual care and list of services | | | | |
| Low risk | Low risk | Low risk | Individual RCT with low attrition (two rigorous follow-ups; N = 2,079–2,088) | Primarily low-income mothers in urban settings (16 sites across the U.S.) |
| Nurse Family Partnership: Elmira trial (Olds et al. 1997) compared to developmental screening and referrals | | | | |
|  |  | Low risk | Individual RCT with low attrition (N = 245) | Primarily first-time mothers in a rural setting (Elmira, NY) |
| Nurse Family Partnership: Denver trial (Olds et al. 2002, 2004) compared to developmental screening and referrals | | | | |
| Low risk |  | Low risk | Individual RCT with low attrition that controlled for chance differences across groups (two rigorous follow-ups; N = 417–427) | Primarily first-time, low-income mothers in an urban setting (Denver, CO) |
| Nurse Family Partnership: Memphis trial (Kitzman et al. 1997, 2000; Olds et al. 2004, 2007, 2010) compared to developmental screening and referrals | | | | |
| Low risk |  | Low risk | Individual RCT with low attrition that controlled for chance differences across groups (five rigorous follow-ups; N = 594–675) | Primarily African America, first-time, low-income mothers in an urban setting (Memphis, TN) |
| Pathways Teen Mother Support Project (McDonell et al. 2007) compared to usual care | | | | |
| Low risk |  | Low risk | Individual RCT with low attrition (three follow-ups; N = 114–138) | Primarily African American, first-time, low-income mothers in a rural setting (SC) |
|  | **Contracept-ive use** | **Repeat pregnancy or birth** | **Study type and explanation of rating** | **Study sample and setting** |
| Project CHARM - Be Proud! Be Responsible! Be Protective! (Koniak-Griffin et al. 2003a) compared to general health promotion information and free condoms | | | | |
|  | Low risk |  | Cluster RCT in schools with low attrition of schools and individuals (N = 497) | Primarily Latino mothers in an urban setting (Los Angeles, CA)^h^ |
| Respeto/Proteger (Koniak-Griffin et al. 2011; Lesser et al. 2009) compared to HIV/AIDs information | | | | |
|  | Moderate |  | Cluster RCT of couples with low attrition of couples, but high attrition for individuals (two rigorous follow-ups; N = 251) | Primarily Latino, first-time, couples in an urban setting (Los Angeles, CA)^i^ |
| Taking Charge (Harris & Franklin 2003) compared to only case management | | | | |
| Moderate |  |  | Individual RCT with high attrition that established balance for education (two rigorous follow-ups; N = 73) | Primarily Latino, first-time mothers in an urban setting (TX)^j^ |
| Teen Options to Prevent Pregnancy (Rotz et al. 2016; Smith et al. 2015) compared to usual care | | | | |
| Low risk | Low risk | Low risk | Individual RCT with low attrition (two rigorous follow-ups; N = 472–493) | Primarily low-income mothers in an urban setting (Columbus, OH)^k^ |
| Teen Parent Demonstration (Kisker et al. 1998) compared to usual care | | | | |
| Low risk |  | Low risk | Individual RCT with low attrition that controlled for chance differences across groups (N = 3,498) | Primarily low-income mothers and fathers in urban settings (Camden, PA; Chicago, IL; Newark, NJ) |
| Teen Parent Home Visitor Services Demonstration (Kelsey et al. 2001) compared to usual welfare-to-work program | | | | |
| Moderate risk | High risk | Moderate | Individual RCT with unknown attrition that established baseline equivalence for education and on SES for repeat pregnancy or birth but that did not establish equivalence for contraceptive use (N = 708) | Primarily first-time, low-income mothers in urban settings (South Chicago, IL; Dayton, OH; Portland, OR) |
| Three generations (Black et al. 2006) compared to usual care | | | | |
|  |  | Low risk | Individual RCT with low attrition (N = 149) | Primarily African American, first-time, low-income mothers in an urban setting (Baltimore, MD) |

Note: RCT = randomized controlled trial, SES = socioeconomic status, CAMI = computer-assisted motivational interviewing.

^a^ The AIM 4 Teen Moms sample was described as low-income, but information on income was not provided so we could not confirm whether 75 percent of the sample was low-income.

^b^ Income was not reported for the CenteringPregnancy Plus HIV prevention: Atlanta/New Haven sample.

^c^ The CenteringPregnancy Plus HIV prevention: New York City sample was from clinical sites that serve predominantly low-resource women, but information on income was not provided so we could not confirm whether 75 percent of the sample was low-income.

^d^ The comparison of a CAMI-only group with a usual care group was also considered rigorous, but we focus on reporting results for the CAMI plus home visiting condition compared to usual care. There were no effects on repeat birth or time to subsequent birth for a CAMI-only group compared with a usual care group.

^e^ In the Dollar-a-Day program, the comparison of the incentive and peer support group to an incentive-only group was also considered rigorous. However, we focus on the comparison of the incentive and peer support group with the peer support-only group because only two participants took part in the peer support-only group, so it is similar to a no-treatment comparison group. Participants in the incentive and peer support group had lower rates of repeat pregnancy than the incentive-only group within 6 months of the prior birth during the program (6 months after enrollment), but there were no effects on repeat pregnancy within 12, 18, and 24 months of the prior birth during the program (12, 18, and 24 months after enrollment).

^f^ Equivalence information for the teen parent sample in Early Head Start was obtained by author query.

^g^ Whether Healthy Families sites in Massachusetts were urban or rural was not reported.

^h^ The Project CHARM sample had an average Hollingshead score of 30, but we could not determine whether at least 75 percent of the sample should be considered low-income. In addition, the study did not report whether mothers were primarily first-time mothers.

^i^ Income was not reported for the Respeto/Proteger sample.

^j^ Income was not reported for the Taking Charge sample.

^k^ The Teen Options to Prevent Pregnancy study did not report whether mothers were primarily first-time mothers.

Appendix Table A.4

*Program details for programs with rigorous evidence*

| Primary intervention (mode)^a^ | Primary setting | Length^b^ and frequency | Type of facilitator^c^ | Program description |
| --- | --- | --- | --- | --- |
| **Programs that improved education outcomes** | | | | |
| Early Head Start (Love et al. 2001, 2002; Vogel et al. 2010)^d^ | | | | |
| Comprehensive family support services (one-on-one and other strategies) | School (early childhood education)  Home | Long  At least weekly | Teacher | Early Head Start programs provide family-centered services for low-income families with very young children to promote the development of the children and to enable the parents to fulfill their roles as parents and move toward self-sufficiency. Programs may be center based, home based, or a combination of the two. In center-based programs, trained teachers provide up to 20 hours of child care and other services each week. In home-based programs, home visitors visit participants weekly. Services are available from pregnancy through the child’s third birthday. |
| Early Intervention Program for Adolescent Mothers (Koniak-Griffin et al. 2000, 2002, 2003b) | | | | |
| Home visiting (one-on-one and other strategies) | Home | Long  At least biweekly | Nurse | The Early Intervention Program for Adolescent Mothers aims to help young mothers gain social competence to manage their worlds more effectively, including providing content on health, life skills, maternal role, and social support systems. Public health nurses visit mothers in their homes approximately 17 times and the mothers attend four group-based “Preparation for Motherhood” classes between pregnancy and the child’s first birthday. |
| Healthy Families America (Jacobs et al. 2016)^e^ | | | | |
| Home visiting (one-on-one) | Home | Medium  At least biweekly | Case worker | Healthy Families America provides comprehensive home visitation services to first-time, highly stressed caregivers who are at risk for child maltreatment. Family Support Workers provide goal setting, curriculum-based activities, individualized family support, health screenings, and linkages to medical and other services as needed during home visits. Family Support Workers visit participants in their homes biweekly during pregnancy and weekly for at least six months following the child’s birth. |
| Learning, Earning, and Parenting Program (Bos & Fellerath 1997) | | | | |
| Case management (one-on-one) | Welfare office | Long  At least monthly | Case worker | The Ohio Learning, Earning, and Parenting (LEAP) program seeks to reduce future welfare receipt by encouraging welfare-eligible teen mothers who do not have a high school degree to complete secondary education. LEAP provides case management services and financial incentives for school enrollment. Mothers are expected to be enrolled in school while they are receiving welfare, and mothers who do not comply are sanctioned. Case managers evaluate teens’ enrollment status monthly, offer guidance, and authorize support services. Adolescents can participate until they receive their high school diploma/GED, no longer receive welfare, or turn 20. |
| New Chance (Quint et al. 1997)^f^ | | | | |
| Educational or employment services (one-on-one and other strategies) | Community based | Long  Daily | Case worker | New Chance, a program for young welfare-eligible mothers who have dropped out of school, aims to improve mothers’ employment potential and parenting skills through curriculum-based courses and individualized case management. The 18-month program offers regular educational and employment services as well as child care, with case managers providing up to an additional 12 months of follow-up. |
| Taking Charge (Harris & Franklin 2003) | | | | |
| Group-based curriculum (small-group) | School | Short  At least weekly | Social worker | Taking Charge is a task-centered, cognitive-behavioral, school-based curriculum that aims to improve problem-focused coping behavior, social problem-solving skills, and school achievement. Participants receive small incentives for participating in the program. The eight-session curriculum is delivered weekly by social workers with a young mother cofacilitator in a school-based setting. |
| **Program that improved education outcomes and reduced repeat pregnancies or births** | | | | |
| Pathways Teen Mother Support Project (McDonell et al. 2007) | | | | |
| Case management (one-on-one and other strategies) | Community based | Long  Variable | Case worker | The Pathways Teen Mother Support Project aims to strengthen mothers’ personal and social competencies, increase support from teens’ immediate and extended family, and increase the role of community social institutions in supporting the teens’ success. The program consists of five voluntary components: case management, family group decision making, mutual assistance peer groups, life skills education, and leadership development activities. Individualized case planning is used to select the services most suited to helping teens meet their goals over two years. |
| **Programs that increased contraceptive use** | | | | |
| AIM 4 Teen Moms (Covington et al. 2015; Rotz et al. 2016)^g^ | | | | |
| Home visiting (one-on-one and other strategies) | Home | Short  At least weekly | Case worker | AIM 4 Teen Moms seeks to reduce rapid repeat pregnancies by helping teen mothers define specific life aspirations, engage in planning to successfully achieve them, and consider the role of contraception in their lives. The 10-week program consists of six one-hour individual sessions, one 90-minute group session at the halfway point, and another 90-minute group session at the end of the program. Trained facilitators deliver the program in teens’ homes and/or community-based locations. |
| CenteringPregnancy Plus HIV prevention (Ickovics et al. 2016; Kershaw et al. 2009)^h^ | | | | |
| Prenatal care (small-group) | Clinic | Medium  At least weekly | Clinician | CenteringPregnancy Plus HIV prevention is a group prenatal care intervention that aims to improve mothers’ reproductive and sexual health outcomes. CenteringPregnancy Plus provides group-based prenatal care that follows the clinical guidelines of the American College of Obstetricians and Gynecologists to groups of 8 to 12  women. Content includes activities to improve HIV knowledge, interpersonal sexual communication, perceived risk, and social norms. A clinician and cofacilitator (nurse or medical assistant) facilitate the sessions. Ten sessions lasting 120 minutes occur prenatally approximately once a week; four of these sessions focus on promoting reproductive health. |
| **Program that increased contraceptive use and reduced pregnancies or births** | | | | |
| Teen Options to Prevent Pregnancy (Smith et al. 2015; Rotz et al. 2016)^i^ | | | | |
| Case management (one-on-one) | Telephone | Long  At least monthly | Nurse | Teen Options to Prevent Pregnancy (TOPP) provides motivational interviewing, contraceptive access, and social service support to help at-risk teen mothers develop and adhere to a birth control plan and prevent rapid repeat pregnancies. Trained nurse educators deliver the program through monthly telephone-based care coordination over an 18-month period. At least one home visit should occur at the start of the intervention, with other home-based follow-ups occurring as needed. |
| **Programs that reduced repeat pregnancies or births** | | | | |
| Computer-Assisted Motivational Intervention plus home visiting (Barnet et al. 2009) | | | | |
| Home visiting (one-on-one) | Home | Long  At least monthly | Case worker | Computer-Assisted Motivational Interviewing (CAMI) uses motivational interviewing to reduce rapid repeat pregnancies to teen mothers. In CAMI sessions, a teen answers questions about her current sexual activity and contraceptive behaviors using a laptop that then provides a summary of risk for pregnancy and sexually transmitted infections. CAMI counselors then conduct 20-minute motivational interviewing sessions. In the CAMI plus home visiting condition, CAMI is paired with home visiting that provides parent training on child development and  case management services to help participants address issues such as housing or child care. For two years, CAMI sessions occur every three months, and home visiting sessions occur biweekly to monthly. |
| Dollar-a-Day Program: Peer support and incentives (Stevens-Simon et al. 1997) | | | | |
| Financial incentive (small-group) | Community based | Long  At least weekly | Case worker | The Dollar-a-Day Program aims to reduce repeat teen pregnancy through providing financial incentives and peer support. Participants come together to collect their financial incentive, share snacks, and talk informally about their concerns about the present and their goals for the future in a supportive, adult-led, peer group environment. Participants receive $1 per day for not getting pregnant and meet weekly for two years. |
| Nurse Family Partnership (Kitzman et al. 1997, 2000; Olds et al. 1997, 2002, 2004, 2007, 2010)^j^ | | | | |
| Home visiting (one-on-one) | Home | Long  Variable | Nurse | The Nurse Family Partnership is designed for first-time, low-income mothers and their children. It aims to improve (1) prenatal health and outcomes, (2) child health and development, and (3) families’ economic  self-sufficiency and/or maternal life course development. It includes one-on-one home visits by a trained public health registered nurse to participating clients. The visits begin early in the woman’s pregnancy (with program enrollment no later than the 28th week of gestation) and conclude when the child is 2 years old. Visits are weekly for the first month after enrollment, biweekly until the child is born, and then as needed. |
| Three Generations (Black et al. 2006) | | | | |
| Home visiting (one-on-one) | Home | Medium  At least biweekly | Case worker | Three Generations is a home-based mentoring intervention to prevent second pregnancies among teen mothers. Drawing on social cognitive theory, the program focuses on interpersonal negation skills, adolescent development, and parenting. The 19-lesson curriculum can be used flexibly to help teens improve their relationship with their own mother, understand their infant’s development, and understand their personal values regarding subsequent pregnancy. Participants receive condoms at every contact. Mentors visit families biweekly from birth until the infant’s first birthday. |
| **Programs that did not improve eligible outcomes** | | | | |
| Cal-Learn: Financial incentives and case management (Mauldon et al. 2000)^k^ | | | | |
| Case  management (one-on-one) | Welfare office | Long  Variable | Case worker | Cal-Learn aims to support pregnant and parenting teenagers receiving welfare to complete high school. The program is mandatory for all custodial parents younger than 19 who do not have a high school diploma or GED who are receiving welfare. Case managers support teens to help them complete their education. They can arrange for  needed services, provide information and referrals, or provide social support as needed. Financial bonuses or penalties of $100 are also implemented four times a year for teens who either show progress in school or fail to make progress according to their report cards, respectively. In addition, teens receive a bonus if they receive their high school diploma or GED. |
| Girl Talk (Katz et al. 2011)^l^ | | | |  |
| Case management (one-on-one and other strategies) | Telephone | Long  At least biweekly | Social worker | Girl Talk provides individual telephone counseling to improve reproductive health planning and motivate teen mothers to delay further childbearing. Using a teen-focused intervention curriculum, counselors work with teens to build knowledge of health risks, develop positive teen attitudes and skills, improve communication, resist peer pressure,  and increase connectedness with families. After an initial face-to-face visit, phone sessions are scheduled to be weekly for the first 6 months and then biweekly for another 12 months. Two-hour group sessions also take place quarterly. |
| Project CHARM - Be Proud! Be Responsible! Be Protective! (Koniak-Griffin et al. 2003a)^m^ | | | | |
| Group-based curriculum (small group) | School | Short  At least biweekly | Nurse | Project Charm uses a modified HIV intervention program “Be Proud! Be Responsible! Be Protective!” to motivate adolescents to make heathy sexual decisions. Educational experiences include group discussions, videos, games, and skill-building activities related to sexual negotiation and condom use. The program includes four two-hour sessions. |
| Respeto/Proteger (Koniak-Griffin et al. 2011; Lesser et al. 2009)^n^ | | | | |
| Group-based curriculum (couple focused) | Community based | Short  At least biweekly | Case worker | Respeto/Proteger: Respecting and Protecting Our Relationships is a culturally rooted, couple-focused and asset-based HIV prevention program for young Latino parents that builds on feelings of paternal protectiveness while integrating cultural teaching as motivation to reduce risky sexual behavior. Each series of classes includes a 12-hour  curriculum intended for small groups of couples. |
| Teen Parent Demonstration (Kisker et al. 1998)^o^ | | | | |
| Educational or employment services (one-on-one) | Welfare office | Long  At least weekly | Case worker | The Teen Parent Demonstration aims to help young welfare-eligible mothers become self-sufficient by helping them set goals related to attaining education, training, and employment. The program requires mothers receiving welfare to participate in initial workshops and then in education, job training, or employment-related activities while  receiving support services through individualized case management delivered by case managers. Participation among mothers is mandatory while they are receiving welfare; mothers who do not comply with planned activities risk losing cash benefits. |
| Teen Parent Home Visitor Services Demonstration (Kelsey et al. 2001)^p^ | | | | |
| Home visiting (one-on-one) | Home | Variable length  At least weekly | Case worker | The Teen Parent Home Visitor Services Demonstration aims to improve low-income teen mothers’ participation rates in required welfare-to-work activities (Job Opportunities and Basic Skills Training Program) and promote greater self-sufficiency. Home visitors provide instruction, support, and other necessary assistance in weekly meetings for up to 30  months as long as the mother is receiving welfare. Mothers who do not comply with home visits risk losing cash benefits. |

*Note*. Program descriptions describe the programs as they were implemented in the eligible studies.

^a^ Programs that provide one-on-one services and other strategies use a number of different approaches. For example, a program may primarily offer one-on-one home visiting but also offer small group parent training sessions.

^b^ Program length definitions are as follows: short programs are fewer than 3 months long, medium programs are 3 to 12 months long, long programs are 13 months or longer.

^c^ A case worker does not have a professional license. Some programs require case workers to have a certain educational qualification, but others do not.

^d^ Early Head Start also rigorously tested and did not find effects for repeat pregnancy or birth.

^e^ Healthy Families America also rigorously tested and did not find effects for repeat pregnancy or birth.

^f^ New Chance also rigorously tested and did not find effects for contraceptive use and repeat pregnancy or birth.

^g^ AIM 4 Teen Moms also rigorously tested education outcomes and did not find effects.

^h^ CenteringPregnancy Plus HIV prevention also rigorously tested and did not find effects for repeat pregnancies or births.

^i^ Teen Options to Prevent Pregnancy also rigorously tested and did not find effects for education.

^j^ Nurse Family Partnership also rigorously tested and did not find effects for education.

^k^ Cal-Learn: Financial incentives and case management rigorously tested and did not find effects for repeat pregnancy or birth.

^l^ Girl Talk rigorously tested and did not find effects for repeat pregnancy or birth.

^m^ Project CHARM - Be Proud! Be Responsible! Be Protective! rigorously tested and did not find effects for contraceptive use.

^n^ Respeto/Proteger rigorously tested and did not find effects for contraceptive use.

^o^ Teen Parent Demonstration rigorously tested and did not find effects for education and repeat pregnancy or birth.

^p^ Teen Parent Home Visitor Services Demonstration rigorously tested and did not find effects for education and repeat pregnancy or birth.
